# Can academic detailing reduce opioid prescriptions in chronic non-cancer pain?

**DOI:** 10.1186/s12875-023-02040-6

**Published:** 2023-03-27

**Authors:** Ketil Arne Espnes, Torunn Hatlen Nøst, Marte Handal, Svetlana O. Skurtveit, Harald C. Langaas

**Affiliations:** 1 KUPP – The Norwegian Academic Detailing Program. Department of Clinical Pharmacology, St. Olavs Hospital, Trondheim University Hospital, P.O.Box 3250 Torgarden, 7006 Trondheim, Norway; 2 Section for Drug Safety, Department of Clinical Pharmacology, St. Olavs Hospital, Trondheim University Hospital, Trondheim, Norway; 3 Department of Mental Health, Faculty of Medicine and Health Sciences, Norwegian University of Technology and Science, Trondheim, Norway; 4 Norwegian Advisory Unit On Complex Symptom Disorders, St. Olavs Hospital, Trondheim University Hospital, Trondheim, Norway; 5 Norwegian Institute of Public Health, Oslo, Norway; 6 Norwegian Centre for Addiction Research (SERAF), Institute of Clinical Medicine, University of Oslo, Oslo, Norway; 7 Regional Medicines Information and Pharmacovigilance Centre (RELIS), Department of Clinical Pharmacology, St. Olavs Hospital, Trondheim University Hospital, Trondheim, Norway

**Keywords:** Chronic non-cancer pain, Academic detailing, Primary care, Opioids

## Abstract

**Background:**

One measure to support optimal opioid prescription is academic detailing (AD) with one-to-one visits by trained professionals (academic detailers) to general practitioners (GPs).

Objective: To investigate the usefulness of AD visits on GPs’ opioid prescribing patterns in Norway, and academic detailers’ experiences with AD visits to GPs on opioid prescription.

**Methods:**

Design: A quantitative registry study on opioid prescriptions and a qualitative focus group interview study with academic detailers.

Participants: For the registry study, municipalities where more than 75% of the GPs had received an AD visit were considered intervention municipalities, whereas in the non-intervention municipalities no GPs had received AD-visits. In the focus groups, academic detailers who had conducted three or more AD-visits were invited to participate.

Intervention: A campaign on opioid prescription with AD visits using a brochure with key messages based on the national guideline for treatment of chronic non-cancer pain and updated evidence on the potential benefits and risks of prescribing opioids. The AD visits in the campaign were planned for 20–25 min in a one-to-one setting in the GP’s office.

Main Measures: The Norwegian Prescription Database (NorPD) was utilized for registry data. Data on amount of drugs dispensed are recoded as Defined Daily Doses (DDDs).

**Results:**

Compared to non-intervention, the intervention resulted in a decrease in the number of prevalent and incident users of opioids and incident users of reimbursed opioids for chronic non-cancer pain in municipalities in Central Norway. The results from the focus group interviews were categorized into the themes: “To get in position”, “Adjusting messages”, “What did the GPs struggle with, in relation to opioid prescription?” and “Did we reach the right recipients with the visits?”.

**Conclusions:**

In Central Norway, the intervention resulted in a desired effect on number of opioid users. According to the academic detailers, the GPs’ length of working experience and familiarity with the topic gave different presumptions for making use of the information presented in the AD-visits.

**Supplementary Information:**

The online version contains supplementary material available at 10.1186/s12875-023-02040-6.

## Background

Internationally, an opioid epidemic has evolved during the two first decades of the twenty-first century. Especially, the use of opioids has increased in North America, Western Europe, and Oceania [1]. Increased prescribed analgesic opioid use has been followed by a dramatic increase in addiction disorders, use of illegal opioids, overdose deaths and even in the number of suicides [2–4]. Long-term opioid treatment is also problematic due to serious adverse effects such as sedation, cognitive impairment and tolerance development, with potentially devastating effects on functional capacity and quality of life [5–7].

The short-term use of opioids for acute and terminal conditions has traditionally been widely accepted. Conversely, there is no strong evidence to support the use of opioids in chronic non-cancer pain conditions, while risks and adverse effects are well established. Long-term use of opioids should therefore usually be avoided for chronic non-cancer pain [5, 8]. Consequently, updated and knowledge-based practice for opioid prescription is important to avoid further development of an opioid epidemic.

One measure to support optimal opioid prescription is academic detailing (AD). The term academic detailing was first introduced by Avorn and Soumerai in 1983 [9], when they showed that prescribers receiving personal educational visits (AD visits) reduced the prescription of target drugs significantly compared to groups that received either specified printed information on the matter only or no specific information beside the standard.

AD visits have shown to be a useful and cost-effective way to improve the quality of decisions made about prescribing drugs, as well as to reduce unnecessary expenses [9]. A systematic review found that AD can be effective, either as a single intervention or as part of a multiple intervention to change the prescriber’s practice [10]. Moreover, AD has been proposed as a potentially effective intervention to address the epidemic of opioid overuse [11]. A recent publication showed considerable alignment between self-reported practice change intentions following academic detailing and actual changes in subsequent opioid prescribing [12], whereas a British study on the effects of an evidence- and theory-informed feedback intervention on opioid prescribing for non-cancer pain in primary care, found that prescribing of strong opioids, total opioid prescriptions, and prescribing in high-risk groups generally fell, although effects lessened after the feedback stopped [13].

In summary, there are several studies that have done assessments on the changes resulting from AD [14, 15]. Most studies have been reported from USA [16], but only a few of them concern opioid prescription. Hence, few studies have reported on usefulness of AD regarding opioid prescription outside USA. Furthermore, there is a lack of knowledge on what professionals who perform AD-visits (academic detailers) experience as widespread challenges in general practitioners’ (GPs’) opioid prescribing for chronic non-cancer pain.

Hence, the aim of the current study was to investigate the usefulness of AD visits on GPs’ opioid prescribing patterns in Norway, and academic detailers’ experiences from AD visits to GPs on opioid prescription.

## Methods

A quantitative registry study on opioid prescriptions in Norway and a qualitative focus group interview study with academic detailers were conducted.

### Intervention: The Norwegian academic detailing campaign on opioid prescription

*KUPP – The Norwegian Academic Detailing Program* is a nationwide initiative in Norway, located at the Regional Medicines and Pharmacovigilance Centers (RELIS) [17–19]. In 2018, KUPP decided to take part in the national effort in reducing opioid use in chronic non-cancer pain.

For this, KUPP initiated a campaign which included the development of a four-page brochure based on the national guideline for treatment of chronic non-cancer pain [20] and updated evidence on the potential benefits and risks of prescribing opioids for these conditions.

The key messages in the campaign were based mainly on an evaluation of the risk for adverse effects, including addiction and overdosing, but also included information on the lack of documented effect of opioids in long-term use. The campaign focused on the most correct use and general risks of opioids, and the main message was as far as possible to avoid opioid use for chronic non-cancer pain. The brochure’s highlighted five key messages to the GPs are presented in Table 1.

**Table 1:** The five key messages highlighted in the brochure given to the GPs

1) The benefit of opioid use in chronic non-cancer pain is not scientifically verified
2) Non-pharmacological interventions are central in patients with chronic non-cancer pain
3) Try non-opioids as first pharmacological intervention in chronic non-cancer pain
4) If starting opioids: Avoid co-medication with other central nervous depressant medications
5) Treatment with opioids should always comply with a set plan and be evaluated at frequent intervals

The AD visits were planned for 20–25 min in a one-to-one setting in the GP’s office. All the academic detailers (experienced pharmacists and consultants or residents in clinical pharmacology) had participated in a three-day training course in AD as well as a one-day training session on this specific campaign. They were all employed at governmentally funded public hospitals. Each visit was designed to run through the key messages, but the academic detailers were trained to emphasize on the topics where they found (pronounced or unpronounced) that the physician had needs for more information, all according to international standards of Academic Detailing [9, 18, 21, 22]. Visits were conducted in September – November 2019.

### The prescription registry data

#### Data source

The Norwegian Prescription Database (NorPD) was utilized for the quantitative registry study. The NorPD contains information on all prescription drugs dispensed from Norwegian pharmacies and about reimbursement of drugs according to diagnosis. Diagnoses are classified by either the 10^th^ edition of the International Classification of Diseases (ICD-10 codes) or the International Classification of Primary Care (ICPC-2 codes) or according to specifically generated reimbursement codes.

In Norway, analgesic opioids are only available via prescription and the NorPD therefore includes information on all dispensed opioids in ambulatory care. Conversely, drugs used by patients in hospitals and other institutions are not included in the NorPD. Opioids can be reimbursed for chronic non-cancer pain using such a specifically generated reimbursement code. By using this code, the prescribing physician states that the patient meets a predefined set of criteria for a disease or medical state resulting in chronic non-cancer pain.

In this study we used filled prescriptions as a proxy for the GPs’ prescriptions. Information about prescriptions that are not filled (primary non-compliance) will not be present in the NorPD [23].

#### Study population

Municipalities where more than 75% of the GPs had received an AD visit were considered intervention municipalities, whereas in the non-intervention municipalities no GPs had received AD-visits. For each municipality defined as an intervention or as a non-intervention municipality we identified the number of patients who had filled at least one prescription for an analgesic opioid (ATC-codes N02A*) and the dispensed amount of opioid during the specified time periods, as well as the number of inhabitants. The academic detailers booked their own meetings, and a large proportion of the visits were done with GPs who had previously received AD visits on other themes. As a result, the municipalities were not randomly decided to be in the intervention or non-intervention group. Due to the lower share of GPs visited in two of Norway’s four health regions, analyses were only possible in two regions, Northern Norway and Central Norway.

#### Data

Drugs in the NorPD are registered according to the WHO’s Anatomical Therapeutic Chemical (ATC) classification system, where analgesic opioids (ATC code N02A) were included into this study. This means that opioids for opioid maintenance therapy (N07BC) and antitussives (R05DA), which are rarely used for analgesia in Norway, were not included.

Data on amount of drugs dispensed are recoded as Defined Daily Doses (DDDs). The definition of DDD is «the assumed average maintenance dose per day for a drug used for its main indication in adults». The DDD for each drug was retrieved from the WHO collaborating centre for drug statistics methodology [24]. This is presented in Table 2.

**Table 2:** DDD (2022) for the analgesic opioids included in the study

ATC code	ATC level name	DDD	Unit	Administration Route
N02AA01	morphine	0.1	g	O
N02AA01	morphine	30	mg	P
N02AA01	morphine	30	mg	R
N02AA05	oxycodone	30	mg	P
N02AA05	oxycodone	75	mg	O
N02AA55	oxycodone and naloxone	75	mg	O
N02AB03	fentanyl	0.6	mg	N
N02AB03	fentanyl	0.6	mg	SL
N02AB03	fentanyl	1.2	mg	TD
N02AE01	buprenorphine	1.2	mg	P
N02AE01	buprenorphine	1.2	mg	SL
N02AE01	buprenorphine	1.2	mg	TD
N02AJ06	codeine and paracetamol^a^			
N02AX02	tramadol	0.3	g	O
N02AX02	tramadol	0.3	g	P
N02AX02	tramadol	0.3	g	R
N02AJ13	tramadol and paracetamol^b^			

*DDD* Defined Daily Dose

Administration Route: *O* Oral, *N* Nasal, *SL* Sublingual, *TD* Transdermal, *R* Rectal, *P* Parenteral

^a^combination of two analgesics; example: 4 pills of 30 mg codeine + 0.4 g paracetamol

^b^combination of two analgesics; example: 4 pills of 37.5 mg tramadol + 0.325 g paracetamol

#### Analysis strategy for registry data

First, we calculated 12 months prevalence (1-year prevalence per 1000) of opioid use before the intervention and 12 months prevalence of opioid use after the intervention in the intervention municipalities. In the non-intervention municipalities we calculated 12 months prevalence (1-year prevalence per 1000) in the same way for the same calendar time period.

Next, we calculated the number of incident users and incidence rate per 1000. An incident user was a person that had at least one opioid prescription dispensed in the study period (before or after intervention), but no opioid prescriptions dispensed the last 365-day period before the respective study periods. Incidence rate was calculated as the number of incident users divided with the municipality population at risk of becoming an analgesic opioid user.

Lastly, we identified opioid users with chronic non-cancer pain utilizing the specific reimbursement code in the NorPD and we calculated the incidence rate of opioid users with reimbursement for chronic non-cancer pain per 1000.

For statistical comparison between the intervention and non-intervention municipalities we used Pearson’s Χ^2^-test.

### The focus group interview study

#### Informants and recruitment

Eligible informants were academic detailers who had carried out a minimum of three one-to-one visits to GPs in this specific campaign. To obtain data that displayed variation in experiences, the aim was to have differences in profession, working region, age, sex, and previous AD-visit experience.

Recruitment was done by the KUPP management. They identified and invited eligible academic detailers from all four regions to participate and passed forward information and consent forms. Those who agreed to participate returned a signed consent to the project and a time for the focus group interviews was set. Recruitment continued until a minimum of five participants from each region were included as this was assumed to provide sufficient data to answer the aim of the study.

#### Data collection and interview guide

Based on the choice of the informants and the Covid-19 regulations, data collection was carried out using the digital platform Zoom (https://zoom.us/). Data were collected in four focus group interviews with 5—6 informants in each group during April and May 2021. The first author THN conducted all focus group interviews, which ranged in duration between 68 and 80 min. All focus group interviews were audio recorded. They were repeatedly listened to by first author THN who took notes and transcribed the most important parts that were used during the analysis process. Notes and reflections were written down immediately after each focus group interview.

A semi-structured interview guide was developed for this study, based on the research question, previous studies, and discussions with persons experienced in AD. The main question was ‘Can you please tell me how it was to carry out the academic detailing visits on opioid prescription?’ This was followed by questions on what, in their experience, was the most talked about challenges with opioid prescriptions among GPs and which topics the GPs brought up during the visits, and whether they believed that the opioid prescription among GPs would change as a result of the AD-visits.

#### Analysis for focus group interviews

The data, including notes, reflections and transcribed interview parts, were analysed using systematic text condensation, which is a descriptive thematic cross-case analysis strategy involving an iterative four-step analysis procedure [25]. In the first step, the aim was to get an overall impression of the data and to identify preliminary themes. In the second step, all focus group interviews were reviewed to identify relevant meaning units. The meaning units were coded, classified and sorted into code groups related to the preliminary themes. In the third step, a systematic abstraction of meaning units within each of the themes was performed, reducing the content into a condensate that maintained the informants’ responses. In the final step, the content of the condensates was synthesised into generalised descriptions and concepts, which are presented in the Results section.

To expose the data for different views and perspectives, preliminary results were discussed several times with researchers experienced in qualitative methods at the university.

## Results

### Results from the registry study (NorPD)

Table 3 shows the number of prevalent and incident users before and after the intervention in Central and Northern Norway, respectively. There was a significant reduction in both prevalent and incident users in Central Norway when we compared intervention and non-intervention municipalities. There was no significant change in Northern Norway.

**Table 3:** Number and proportion (1-year prevalence and last year first incidence) of users of prescription opioids before and after intervention in municipalities in Central and Northern Norway

	Before intervention			After intervention				
	Number of users (1-year prevalence per 1000)	Total number of DDD per user per year (mean)	Number of incident users (1-year incidence per 1000)	Number of users (1-year prevalence per 1000)	Total number of DDD per user per year (mean)	Number of incident users (1-year incidence per 1000)	**P** number of users intervention versus non intervention	**P** number of incident users intervention versus non intervention
**Central Norway**								
municipalities with intervention	17,168 (98)	53.5	10 166 (64)	16,642 (94)	54.4	9630 (60)		
p							0.009	0.002
municipalities without intervention	22,746 (110)	54.4	13,016 (71)	22,894 (111)	55.3	13,076 (72)		
**Northern Norway**								
municipalities with intervention	11,187 (113)	56.0	6078 (68)	11,143 (111)	56.8	5934 (66)		
p							0.376	0.342
municipalities without intervention	20,226 (110)	63.0	10,802 (66)	19,848 (109)	62.8	10,317 (64)		

*DDD* Defined Daily Dose

The main intervention effect was seen in the number of incident users who received reimbursed opioids for chronic non-cancer pain in Central Norway (Table 4), whereas this was not seen in Northern Norway.

**Table 4:** Number and proportion (1-year prevalence and last year first incidence) of chronic pain patients' prescriptions of reimbursed opioids^a^ before and after intervention in municipalities in Central and Northern Norway

	Before intervention					After Intervention						
	Number of users (chronic pain)	1-year prevalence per 1000 (chronic pain)	Total number of DDD per user per year (mean)	Number of incident individuals (chronic pain)	1-year incidence per 1000 (chronic pain)	Number of users (chronic pain)	1-year prevalence per 1000 (chronic pain)	Total number of DDD per user per year (mean)	Number of incident individuals (chronic pain)	1-year incidence per 1000 (chronic pain)	P number of users intervention versus non intervention	P number of incident users intervention versus non intervention
**Central Norway**												
municipalities with intervention	960	5,4	207	289	0.8	919	5,2	220	186	0.5		
p											0.171	0.007
municipalities without intervention	919	4,5	193	278	0.7	964	4,7	191	255	0.6		
**Northern Norway**												
municipalities with intervention	289	2,9	185	93	0.5	313	3,1	166	92	0.5		
p											0.637	0.952
municipalities without intervention	576	3,1	206	188	0.5	592	3,2	193	181	0.5		

*DDD* Defined Daily Dose

^a^reimbursement for chronic pain

### Results from the focus group interview study

In total, 21 informants participated in the focus groups, 13 women and eight men, with a mean age of 45 years (range 29—67 years). Nine of the informants were pharmacists and 12 were physicians. The majority of the informants had worked on other AD campaigns before, on medication use for type 2 diabetes mellitus (T2DM), NSAIDs and/or antibiotics.

Overall, the campaign on opioid prescription had been well received and even said to be long-awaited by many GPs. In the informants’ experience, the GPs’ length of working experience and familiarity with the topic gave different presumptions for making use of the content in the brochure. For instance, it varied how many opioid users GPs had on their list, depending on such as the age mix in their patient population. As academic detailers they therefore emphasised to adjust the visits to the individual GP’s needs and questions. The results from the qualitative part were categorized into the themes: “To get in position”, “Adjusting messages”, “What did the GPs struggle with, in relation to opioid prescription?” and “Did we reach the right recipients with the visits?”.

#### To get in position

With the AD-visits on opioid prescription, informants said it had been important to position themselves so that GPs did not feel attacked or were put on the defensive because that would make it difficult to come through with their messages. One way of doing this was to emphasise that they recognised and understood that opioid prescription could be difficult, and that GPs had various reasons for prescribing opioids.

*So, it was about communicating what was in the brochure so that they would not take it as an attack or a reprimand and rather show understanding for that this is a difficult topic*.

Academic detailers who previously had worked as GPs themselves said it probably was easier for them to understand the GPs’ struggles because they had experienced similar challenges themselves.

#### Adjusting messages

As basis for the AD-visits, the informants used the key messages in the brochure. Still, the informants found this brochure to differ from other AD-campaign materials they had used, because it did not really present a clear solution to the GPs challenges, but rather presented alternatives for the GPs to try. For many of the informants the non-pharmacological interventions as alternatives to opioid prescription represented unknown territory, because their expertise was in pharmacological treatment. One example talked about was motivational interviewing, on which some informants did not have any expertise. One way of working around this was to just briefly mention the non-pharmacological interventions and otherwise use the visit to go through effects and side effects of medications, and discussions on specific medications.

The informants spoke about adjusting their messages to the GPs by passing on that if adding a new patient to the list of opioid users could be avoided, that was good enough. They found that GPs often fell more at ease when that was said, in specific GPs who were following patients initiated on opioids by other prescribers and therefore felt obliged to renew prescriptions. Concentrating on avoiding new users was thus perceived to be more manageable, among other things because that was something GPs themselves could control.

*You are met with a, a bit of despair because they [GPs] do not have time to deal with everything. And then I experienced that many were relieved when I said that if you manage not to start new ones, then, that is the key message for this visit.*

#### What did the GPs struggle with, in relation to opioid prescription?

In the informants’ experience, one prominent dilemma spoken of by many GPs was how to make changes in opioid prescriptions while maintaining a good doctor-patient relationship.

*They had tried to approach it [opioid reduction] in different ways, but it was especially the maintenance of a good relationship with the patient that I perceived could be in conflict with a correct use of drugs, theoretically speaking.*

The informants in some way or another had mentioned the non-pharmacological interventions from the brochure, although they found the interventions to be outside their area of expertise. However, they found that GPs not necessarily had access to these alternative interventions for their patients and therefore did not find them to be realistic options. For instance, GPs had talked about long waiting lists for psychomotor physiotherapy and a lack of acceptance and commitment therapy (ACT) and mindfulness interventions in their community.

#### Did we reach the right recipients with the visits?

The informants discussed whether they had reached the right recipients of the campaign because even though the GP was the one often left with questions on opioid prescriptions, other services were regularly involved as well. For instance, several informants had met GPs who talked about their experiences with pain clinics.

*That they who sort of should be, one to refer and to get good help from. But there were several who said that this had not worked. That they [patients] had come back with perhaps more medication and there had been suggestions about things that did not suit the patient, and which might actually have contributed to making the situation worse.*

Moreover, GPs frequently had talked about how patients returning to their office after surgery had been given more opioids than recommended for postoperative pain medication. When they came to their GP, they had already used opioids for some time and wanted a refill. Although GPs not automatically renewed the prescription, they missed that colleagues at the hospital also worked on reducing opioid use by prescribing smaller number of opioids. Hence, the informants suggested that the campaign also could be presented for physicians at the hospital, and moreover, that a similar campaign could be designed for patients to inform about when and when not opioid prescription is a recommended and suitable treatment.

## Discussion

The AD campaign on opioid prescription had been well received by the visited GPs. The non-intervention municipalities in Central Norway showed a slight increase in prevalence of opioids use, similar to what have been shown in the national totals for many years [23, 26, 27]. In the intervention municipalities, there was a decrease in the total number of prevalent and incident users of opioids in Central Norway, but not in Northern Norway. One of the reasons probably being that the number of opioid users, and particularly those using reimbursed opioids for chronic non-cancer pain, before the intervention were lower in Northern Norway [28].

We did only study a one-year period after the intervention. Tapering of opioid analgesic treatment might be a slow process that takes time and changes might not have been discovered during the first year.

The findings are in line with a British study [13], which found that prescribing of strong opioids, total opioid prescriptions, and prescribing in high-risk groups generally fell in intervention practices and rose in control practices. The intervention in the British study included feedback to the prescribers on their prescribing of opioids. Although this separates the British intervention from the AD-visits, the similarities of the content in the messages to the prescribers in the interventions gives support to that updated evidence-and theory-based information on opioid prescription is useful for prescribers in primary care.

Midboe et. al [16] highlight one-on-one sessions and provider networking as two of five key lessons important to gain success performing AD interventions addressing the opioid epidemic. The AD-visits in the current study were performed one-on-one, and most of the providers visited had been part of previous AD campaigns and were thus familiar with both KUPP and the academic detailers. Midboe et. al [16] also suggest that training detailers in motivational interviewing (MI) in general is helpful. The academic detailers in the current study were not specifically trained in MI, but they all had taken part in a one-day training course specially designed for the campaign. Our study can add that academic detailers might need more knowledge on the various treatment options that are actually available for the different GPs they visit, including MI. Notably, further research should explore the GPs experiences and views on this as there is a lack of knowledge on this matter from their perspective.

According to the academic detailers not all suggested non-pharmacological treatment options presented in the campaign were considered as relevant or available by the GPs they had visited. This implies that, even though the suggested alternatives to opioids were considered well suited for chronic non-cancer pain patients, they were not necessarily perceived as available for the GPs and their patients. With regards to the finding in the registry study, it might be that a lack of available non-pharmacological treatment methods could be a possible explanation for the difference in changes between Central and Northern Norway, understood as a difference in available options to opioid prescription between Central and Northern parts of Norway.

A relevant reason for that the decrease in total numbers of users were less marked than the fall in new (incident) users, is that, due to pharmacodynamical effects of opioids, it will be easier not to recruit new users than to discontinue treatment in established opioid users [29, 30]. This hypothesis is supported by the qualitative finding on the academic detailers’ practice of emphasising that a main message in the campaign was to avoid new opioid users. In addition, the number of patients with chronic non-cancer pain diagnoses has increased steadily the last years, so achieving a non-rise in the number of opioid prescriptions may well represent an improvement when compared to the steady rise the last years [26, 27].

Because a patient’s opioid use includes prescriptions not only from the patient’s GP but also from hospital doctors, a concern among the academic detailers was about having only GPs as the target group for the campaign. Hence, a question raised was whether a similar campaign also should have been offered to hospital prescribers. The effect pain centres and hospital doctors may have on opioid prescriptions has recently been addressed in the new Norwegian National guidelines on prescribing restricted drugs [31], advising only to prescribe small amounts of opioid analgesics before the patients are referred back to their GP for further treatment. From the current study, it can be added that when planning future AD campaigns, one should consider how to bring the same message across to other suited receivers, such as hospital prescribers as well as to patients.

### Strengths and limitations

A major strength of this study is the combined approach with qualitative and quantitative design. Moreover, utilization of registry data excludes possibility of recall bias, and the nature of the registry allow for identification of control municipalities in the quantitative part of the study.

There are some noteworthy limitations. By interviewing the academic detailers, we got focus group participants that had gathered experience from several visits, whereas GPs could have just accounted for their own single visit.

The sampling strategy for the focus groups could have led to a biased sample as the informants were initially identified by the KUPP management. Nevertheless, the sample showed variations as planned. Transcribing only the most important parts from the audio recordings of the focus group interviews can potentially lead to selection bias and influence the results. However, the audio recordings and notes written after each focus group interview were actively used throughout the analysis process to minimize the bias.

Furthermore, data from the NorPD only contains filled prescriptions, i.e. prescriptions where the patients have had their medications dispensed from a pharmacy. Hence, prescribed, non-filled prescriptions are not a part of the NorPD-data. The number of non-filled prescriptions, where a change would reflect a change in patients’ behavior and not in the GPs’ prescription behavior, are most likely to be the same before and after the intervention, and will thus not have influenced our results.

The municipalities were not randomly assigned to receive AD visits or not. The booking process were likely to select GPs that were positive to receive visits. We do not know if the GPs in the intervention municipalities differ from those in the non-intervention municipalities. Since we are looking at change within the different municipalities, we consider this to be acceptable, but if the GPs who were positive to receive visits were more interested and willing to make changes in their prescription practice, this could have influenced the results.

Due to regulatory limitations in the NorPD, the results do not include any changes in prescriptions for other analgesics (e.g., NSAIDs or paracetamol (acetaminophen)) or individual changes in prescribing as we used municipalities as the study unit. Hence, we are not able to compare individual self-reported intention of practice change with the actual change in the individual prescriber’s practice as done by Saffore et al. [12].

Many studies on the use of opioids benchmark their data using morphine/opioid milligram equivalents (MME or OME) as a measure to standardize opioid doses and quantities across agents. However, that was not a possibility within the datasets used in this study.

Using DDDs, as we have done in this study, could be a limitation if the distribution of strong and weak opioids varied in the two time periods that were compared and between the intervention and the control groups. This is because the DDD value of a weak opioid represents a lower analgesic effect than a DDD of a strong opioid since the DDD is a technical value assigned according to indication. Weak opioids are indicated for weak and moderate pain while strong opioids are indicated for strong pain. Since the time period studied was relatively short, and all municipalities were in Norway, it is not probable that there should be large changes in the distribution of prescriptions of weak and strong opioids. Moreover, as this is not a randomized controlled study, other campaigns or messages released during the same period might have influenced the results.

## Conclusions

In the intervention municipalities, we found a reduction in the number of prevalent and incident opioid analgesic users, and most prominent in incident users who received reimbursed opioids for chronic non-cancer pain in Central Norway.

The GPs’ length of working experience and familiarity with the topic gave different presumptions for making use of the information presented in the AD-visits. When planning future AD campaigns, one should consider how to bring the same message across to other suited receivers, such as hospital prescribers, and also to patients.

## Supplementary Information

**Additional file 1. **Interview guide.

## Data Availability

The data that support the quantitative findings in this study are available from NorPD, a national prescription database run by The Norwegian Institute of Public Health. Restrictions apply to the availability of these data, which were used under license of the current study, and so are not publicly available. Data are available from the authors upon reasonable request and with permission of The Norwegian Institute of Public Health. The intervention material can be made available from the authors upon reasonable request. Due to regulations of the Norwegian Social Science Data Services, NSD, the anonymity of the informants in the qualitative study must be secured. In the raw data, it is possible to identify the informants, and restrictions therefore apply to the availability of these data. Reasonable requests concerning these data can be sent to the corresponding author.

## Abbreviations

AD: Academic Detailing
GP: General Practitioner
KUPP: The Norwegian Academic Detailing Program
RELIS: Regional Medicines and Pharmacovigilance Centers
NorPD: The Norwegian Prescription Database
DDD: Defined Daily Dose
ATC: WHO’s Anatomical Therapeutic Chemical classification
MME: Morphine milligram equivalents
OME: Opioid milligram equivalents
