# Supplementary material for: Can academic detailing reduce opioid prescriptions in chronic non-cancer pain?
Source: BMC Prim Care. 2023 Mar 27;24:84. doi: 10.1186/s12875-023-02040-6 (PMC10042395; doi:10.1186/s12875-023-02040-6)
Supplement: Supplementary file 1 — Additional file 1. Interview guide. [file 12875_2023_2040_MOESM1_ESM.docx]

# Interview guide

In this interview I will ask you about two main topics. One is about how you experienced one-on-one visits to GPs in the opioid campaign, and the other is, based on your many visits to GPs, what you see as barriers and promoters for better prescribing of opioids for long-term non -malignant pain.

Topic 1

Can you tell about what it was like to carry out the one-on-one visits to the GPs in this KUPP campaign?

Topic 2

Can you tell about the topics that GPs brought up during the visits?

What, in your experience, was the most talked about challenges with opioid prescriptions among GPs?

Do you believe that the opioid prescription among GPs will change as a result of the AD-visits?

Finally, is there anything you want to add?
